# The Influence of Aging Attitudes on the Relationship Between Loneliness and Perceived Health

**DOI:** 10.1093/geroni/igaa057.1048

**Published:** 2020-12-16

**Authors:** Kate Kondolf, Zoë Shelton, Silvia Sörensen

**Affiliations:** University of Rochester, Rochester, New York, United States

## Abstract

Loneliness has negative implications for both psychological and physical wellbeing. Age-related impairments further limit social ties, making older adults with vision loss more susceptible to loneliness. Negative age stereotypes directed at the self over the course of one’s personal aging process (Levy, 2009) have harmful effects on cardiovascular health (Levy et al., 2009), engagement in health behaviors (Stewart et al., 2012), longevity (Sun et al., 2017), and psychological well-being. Feelings of loneliness are strongest among individuals who believe that loneliness is a part of aging (Pikhartova et al., 2015). Although loneliness and aging attitudes are both closely linked to health, their interplay has not been investigated within older adult populations. We hypothesized that attitudes about aging would increase as a result of loneliness and thus help explain the relationship between loneliness and perceived health. This study used baseline data from an intervention study of older adults with Macular Degeneration (N=224, aged 60-96, 63.4% female, 20% low-income). Measures: 8-item UCLA Loneliness Scale, Attitudes toward Own Aging scale (ATOA for self-stereotypes, Lawton, 1975), One-item self-reported health. Results: Linear regression showed significant relationships between loneliness and health (β=-.145, p<.05), loneliness and ATOA (β=.32, p<.001), and self-stereotypes and health (β=-.45, p<.001). Adding ATOA to the model regressing health on loneliness rendered the direct effect of loneliness on health non-significant (β=-.014, p=.833), suggesting a mediator effect. Analyses controlling for age, gender, and education yielded comparable results, with the explained variance increasing from 5% (demographics and loneliness) to almost 24% (adding ATOA as mediator).

